# Effects of Combined Application of Organic and Chemical Fertilizers on Nutrient Accumulation and Utilization Efficiency of Alfalfa

**DOI:** 10.3390/plants15182785

**Published:** 2026-09-11

**Authors:** Lan Wang, Qi Wang, Xuerong Ma, Zhuang Xue, An Yan, Shuai Shao

**Affiliations:** 1 College of Grassland Science, Xinjiang Agricultural University, Urumqi 830052, China; 15530221751@163.com (Q.W.); mxr01012023@163.com (X.M.); 18834016151@163.com (Z.X.); yanan@xjau.edu.cn (A.Y.); shaoshuai828508@163.com (S.S.); 2 Xinjiang Key Laboratory of Grassland Resources and Ecology, Urumqi 830052, China; 3 Postdoctoral Research Station in Grassland Resources and Ecology, Urumqi 830052, China

**Keywords:** alfalfa, organic fertilization, nutrient accumulation, nutrient use efficiency

## Abstract

Global demand for alfalfa, a vital forage resource for ruminants, is growing. Currently, ruminant production is expanding rapidly in parts of Asia and Africa, further driving demand for high-quality alfalfa forage. Nevertheless, long-term indiscriminate fertilization and imbalanced nutrient input ratios in alfalfa production not only lower fertilizer use efficiency and crop productivity but also induce a range of soil degradation and environmental issues, thereby constraining the sustainable development of the forage-livestock industry. In this study, the alfalfa cultivar ‘Xinmu No. 4’ (*Medicago sativa cv.* ‘Xinmu No. 4’) was used as the experimental material. A two-year field block experiment was performed to investigate the effects of different organic–inorganic fertilizer ratios on the nutrient use efficiency of alfalfa, with six fertilization treatments: CM0 (100% cow manure), CM1 (75% cow manure + 25% chemical fertilizer), CM2 (50% cow manure + 50% chemical fertilizer), CM3 (25% cow manure + 75% chemical fertilizer), CM4 (100% chemical fertilizer), and CK (no fertilization application). The results demonstrated that the CM3 treatment (25% cow manure combined with 75% chemical fertilizer) significantly enhanced nitrogen, phosphorus, and potassium accumulation in alfalfa relative to the CK, sole cow manure (CM0), and sole chemical fertilizer (CM4) treatments (*p* < 0.05). Additionally, plant nutrient accumulation in 2025 was markedly higher than that in 2024 across treatments (*p* < 0.05). Combined organic and inorganic fertilization substantially improved the agronomic use efficiency of nitrogen, phosphorus, and potassium in alfalfa, with the CM3 ratio exhibiting the most pronounced beneficial effect. For alfalfa cultivation in Xinjiang, the combined application of 25% cow manure and 75% chemical fertilizer is recommended to optimize nutrient accumulation and utilization efficiency, ultimately boosting alfalfa yield and quality.

## 1. Introduction

Driven by the large-scale expansion of China’s livestock sector, the nationwide demand for forage crops has risen continuously over recent years [1]. To pursue high forage yields and greater economic benefits, growers typically adopt long-term and excessive chemical fertilizer application in practical forage cultivation, resulting in a year-on-year increase in fertilizer inputs. This extensive management practice, featured by long-term blind chemical fertilization and imbalanced nutrient supply, causes substantial fertilizer resource waste and pronounced reductions in nutrient use efficiency, alongside progressive soil acidification. This forms a vicious cycle characterized by high input, low output, severe resource loss, and low utilization efficiency [2,3,4]. Such unsustainable cultivation practices not only decrease crop productivity and economic returns but also aggravate ecological degradation, including soil compaction, secondary salinization, and groundwater contamination. These adverse outcomes substantially disrupt the ecological stability of regional forage-livestock systems and impede the green and sustainable development of forage and livestock industries [5,6].

Alfalfa (*Medicago sativa cv.* L.), a perennial leguminous forage renowned as the “King of Forages”, ranks among the world’s most extensively cultivated forage crops owing to its high biomass yield, superior nutritional quality, wide ecological adaptability, abundant nutrient contents, and robust symbiotic nitrogen fixation capacity. This species also constitutes a core leguminous forage across Northwest China. Its deep, well-developed root system and symbiotic nitrogen-fixing capability deliver premium feed resources for livestock production while exerting irreplaceable functions in ameliorating soil physical structure, boosting soil fertility, and sustaining the stability of grassland ecosystems [7,8,9]. Nutrient uptake, accumulation and use efficiency serve as core metrics for evaluating alfalfa growth performance, nutritional quality and rationality of fertilization regimes, and directly govern forage productivity and economic returns [10,11]. Nitrogen, phosphorus and potassium represent primary macronutrients indispensable for plant growth and development. Their uptake, partitioning, translocation, transformation and use efficiency are jointly modulated by cultivar traits, climatic factors and soil conditions, and tightly coupled with fertilization regimes [12,13,14].

In crop cultivation, sole chemical fertilization enables rapid yield responses but frequently induces adverse outcomes, including soil compaction, nutrient imbalance, and nitrogen loss [15,16]. As an eco-friendly alternative to synthetic fertilizers, organic fertilizers contain abundant bioactive substances and trace elements. They sustain slow nutrient release, rejuvenate soil conditions, improve fertilizer use efficiency, and optimize soil physicochemical properties. Furthermore, organic fertilization enhances soil water and nutrient retention capacity, provides carbon sources and energy for soil microorganisms, and improves the activity and stability of soil microbial communities, ultimately promoting soil fertility and soil health [17,18,19,20,21]. Nevertheless, sole organic fertilization ameliorates soil quality and boosts soil buffering capacity, yet it suffers critical limitations including sluggish nutrient mineralization and poor seasonal nutrient use efficiency [22,23]. Integrated organic–inorganic fertilization reconciles the provision of readily available nutrients and long-term soil amelioration, aligning nutrient supply with the periodic nutrient requirements of alfalfa. This strategy represents a pivotal fertilization regime to elevate crop nutrient use efficiency and mitigate nutrient losses [24]. Prior research has demonstrated that a 30% reduction in synthetic fertilizer input combined with organic amendments constitutes an effective fertilization strategy to boost forage quality and sustain high alfalfa yields in sandy soils [25].

Extensive empirical research has demonstrated that partial replacement of synthetic fertilizers with organic fertilizers is an effective strategy for improving soil nutrient status, enhancing nutrient bioavailability, and sustaining crop productivity. Ren et al. [26] reported that partial substitution of synthetic fertilizers with organic fertilizers increased the total soil nutrient pool and enhanced nutrient bioavailability, thereby increasing grain yield in maize (*Zea mays* L.). Wang et al. [27] confirmed that the combined application of organic fertilizers with phosphorus and potassium fertilizers significantly improved the nutritional quality of alfalfa grown in low-yielding farmland. Pei et al. [28] reported that in rainfed dryland wheat fields in the hilly regions of southern Shanxi Province, a fertilization regime involving organic fertilizer substitution for synthetic fertilizers effectively increased grain yield and quality of wheat (*Triticum aestivum* L.). Lu et al. [29] demonstrated that an organic nitrogen substitution rate of 18–24% increased soil available nutrient content, improved fertilizer use efficiency, and maintained stable, high crop yields. Symanowicz and Skorupka [30] reported that the combined application of nitrogen (N), phosphorus (P), and potassium (K) with iron (Fe) and molybdenum (Mo), each at 0.5 kg·hm^−2^ (designated NPKFe_1_Mo_1_), increased soil nitrogenase activity in alkali-tolerant alfalfa to 563.5 nmol C_2_H_4_, with model-estimated biological nitrogen fixation reaching 618 kg·hm^−2^. Compared with the unfertilized control, this treatment significantly increased alfalfa dry matter yield and nitrogen removal, whereas plant nitrogen concentration remained unaffected. Further increases in Fe and Mo application rates did not confer additional yield benefits.

To date, most existing studies have primarily focused on food and cash crops. In contrast, systematic investigations regarding the accumulation, distribution, and use efficiency of nitrogen, phosphorus, and potassium in continuously cropped alfalfa under irrigated oasis conditions in Northern Xinjiang remain limited, particularly for fertilization regimes involving different cow manure and synthetic fertilizer ratios. Furthermore, the differential responses of agronomic and physiological nutrient use efficiency in alfalfa to varied organic–inorganic fertilization ratios, as well as the underlying regulatory mechanisms, remain poorly elucidated [31].

Using ‘Xinmu No. 4’ alfalfa (*Medicago sativa cv.* ‘Xinmu No. 4’) as the experimental cultivar, this study investigated the effects of different ratios of cow manure to chemical fertilizer on N, P, and K accumulation and use efficiency in alfalfa. We further analyzed interannual variation in nutrient uptake under these fertilization regimes and identified the optimal organic fertilizer substitution ratio that balances high yield with efficient nutrient utilization. We hypothesized that an appropriate combination of cow manure and chemical fertilizer would improve soil nutrient supply characteristics and mitigate the limitations of sole chemical fertilization (low nutrient use efficiency) and sole organic fertilization (slow nutrient release). Specifically, we predicted that a moderate substitution treatment (25% cow manure + 75% chemical fertilizer) would enhance N, P, and K accumulation and use efficiency, thereby simultaneously increasing alfalfa yield and nutrient utilization. This study aims to provide a theoretical basis and technical support for efficient fertilization, improved nutrient use efficiency, and sustainable alfalfa forage production in the arid regions of Xinjiang.

## 2. Results

### 2.1. Nutrient Use Efficiency

#### 2.1.1. Nitrogen Accumulation and Nitrogen Utilization

**Figure 1:**
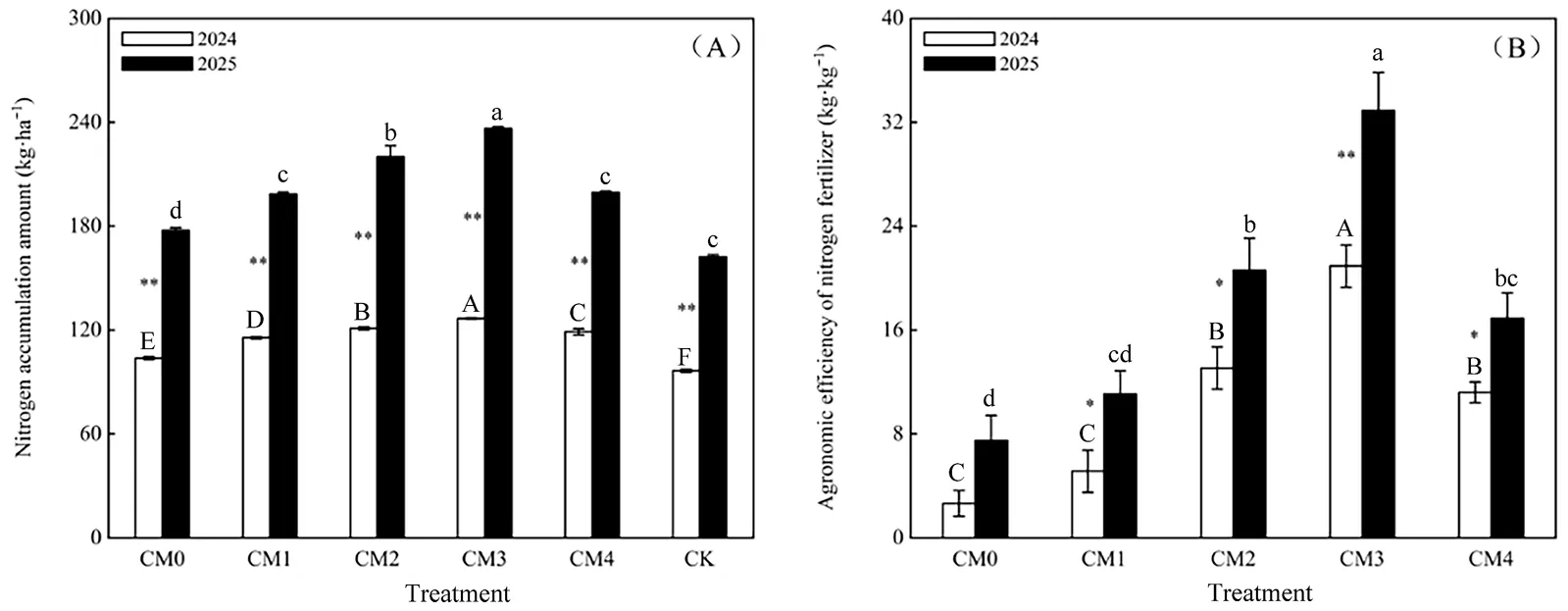
Plant nitrogen accumulation and agronomic nitrogen use efficiency of alfalfa under different fertilizer combinations. Figure (**A**) illustrates nitrogen accumulation, while Figure (**B**) presents nitrogen fertilizer agronomic use efficiency. Different uppercase letters indicate significant differences among different treatments in 2024 at the *p* < 0.05 level. Different lowercase letters indicate significant differences among different treatments in 2025 at the *p* < 0.05 level. * Significant difference for the same treatment between years at the *p* < 0.05 level; ** extremely significant difference for the same treatment between different years at the *p* < 0.01 level.

Varying ratios of organic to synthetic fertilizers differentially affected total N accumulation and agronomic N use efficiency (ANUE) in alfalfa. As shown in Figure 1A, N accumulation was significantly higher under CM3 than under all other treatments in both years; CM2 significantly exceeded CM4, whereas CK exhibited the lowest values. Across all treatments, N accumulation increased highly significantly in 2025 relative to 2024 (*p* < 0.01). As shown in Figure 1B, ANUE was highest under CM3 in both years; no significant difference was detected between CM2 and CM4, although both exceeded CM1, and CM0 exhibited the lowest value, although it did not differ significantly from CM1 in either year. Relative to 2024, ANUE under CM1, CM2, and CM4 increased significantly in 2025 (*p* < 0.05), whereas CM3 showed a highly significant increase (*p* < 0.01).

In both 2024 and 2025, N accumulation and agronomic N use efficiency (ANUE) in alfalfa increased initially and then declined with increasing organic fertilizer substitution ratio (Table 1). In 2024, N accumulation in both cuttings was significantly higher under CM3 than under all other treatments (*p* < 0.05). In the first cutting, no significant difference was observed between CM1 and CM4, whereas CK exhibited the lowest N accumulation and differed significantly from the other treatments (*p* < 0.05). ANUE was highest under CM3 for both cuttings; no significant difference was detected between CM0 and CM1 or between CM2 and CM4. In 2025, nitrogen accumulation in alfalfa plants across four cuttings exhibited an overall downward trend with increasing cutting number. At the third cutting, CM2 and CM3 recorded the highest values; CM1 and CM4 did not differ significantly from each other, but both were significantly higher than CM0 and CK. Nitrogen accumulation under all fertilization treatments was generally higher than under CK, with most CM1–CM4 treatments reaching statistical significance. Agronomic nitrogen use efficiency across all four cuttings was highest under CM3, although at the second cutting CM3 did not differ significantly from CM1 or CM2.

#### 2.1.2. Phosphorus Accumulation and Phosphorus Use Efficiency

**Figure 2:**
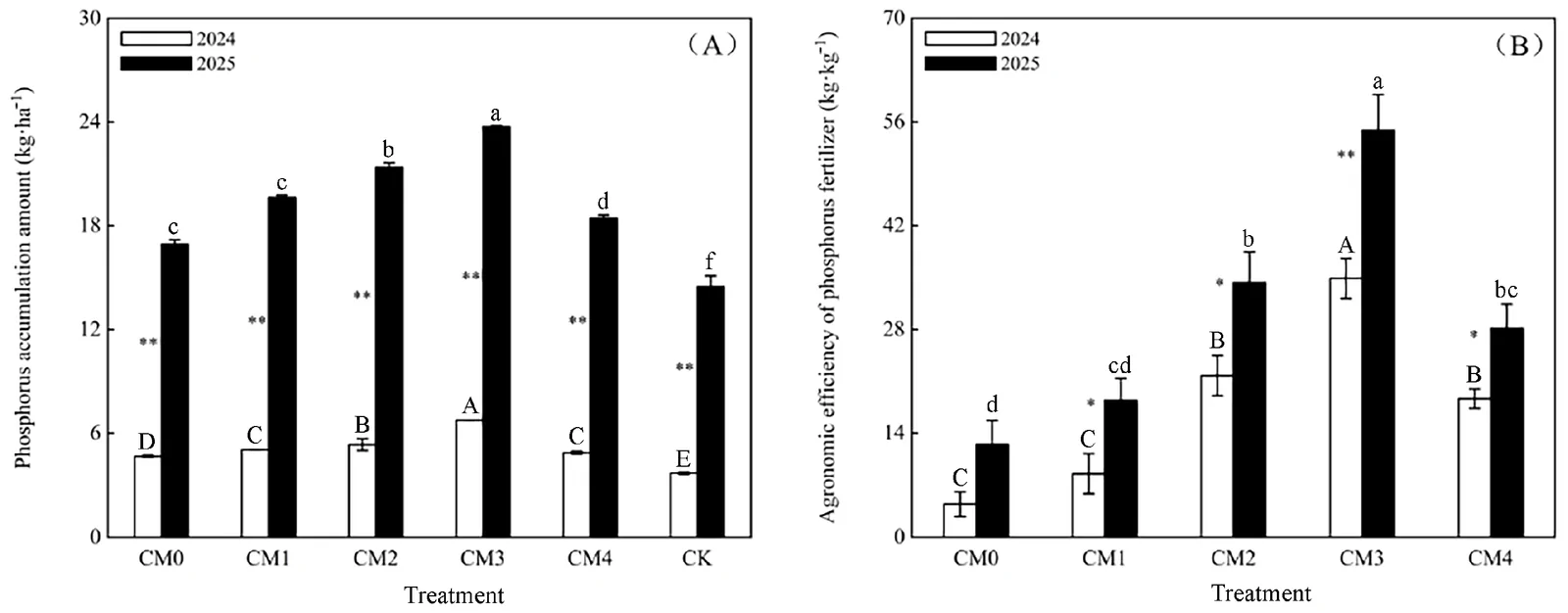
Plant phosphorus accumulation and agronomic phosphorus use efficiency of alfalfa under different fertilizer combinations. Figure (**A**) illustrates phosphorus accumulation, while Figure (**B**) presents the agronomic use efficiency of phosphorus fertilizer. Different uppercase letters indicate significant differences among different treatments in 2024 at the *p* < 0.05 level. Different lowercase letters indicate significant differences among different treatments in 2025 at the *p* < 0.05 level. * Significant difference for the same treatment between years at the *p* < 0.05 level; ** extremely significant difference for the same treatment between different years at the *p* < 0.01 level.

Varying ratios of organic to synthetic fertilizers differentially affected P accumulation and agronomic P use efficiency (APUE) in alfalfa. As shown in Figure 2A, P accumulation was significantly higher under CM3 than under all other treatments in both years; in 2024, no significant difference was observed between CM1 and CM4, whereas CK exhibited the lowest values. Across all treatments, P accumulation increased highly significantly in 2025 relative to 2024 (*p* < 0.01). As shown in Figure 2B, APUE was highest under CM3 in both years; no significant difference was detected between CM2 and CM4, whereas CK exhibited the lowest values. Relative to 2024, APUE in 2025 increased significantly under CM1, CM2, and CM4 (*p* < 0.05) and highly significantly under CM3 (*p* < 0.01), whereas no significant year-to-year difference was observed for CM0.

In both 2024 and 2025, P accumulation and agronomic P use efficiency (APUE) in alfalfa increased initially and then declined with increasing organic fertilizer substitution ratio (Table 2). In 2024, P accumulation in both cuttings followed a consistent trend: it was significantly higher under CM3 than under all other treatments, no significant difference was observed between CM1 and CM4, and CK exhibited the lowest values, differing significantly from the other treatments (*p* < 0.05). APUE in 2024 was highest under CM3 for both cuttings; no significant difference was detected between CM0 and CM1 or between CM2 and CM4. In 2025, P accumulation across the four cuttings was significantly higher under CM3 than under all other treatments, whereas CK exhibited the lowest values and differed significantly from the other treatments (*p* < 0.05). Agronomic phosphorus use efficiency was highest under CM3 across all four cuttings, although at the second cutting CM3 did not differ significantly from CM1 or CM2.

#### 2.1.3. Potassium Accumulation and Potassium Use Efficiency

**Figure 3:**
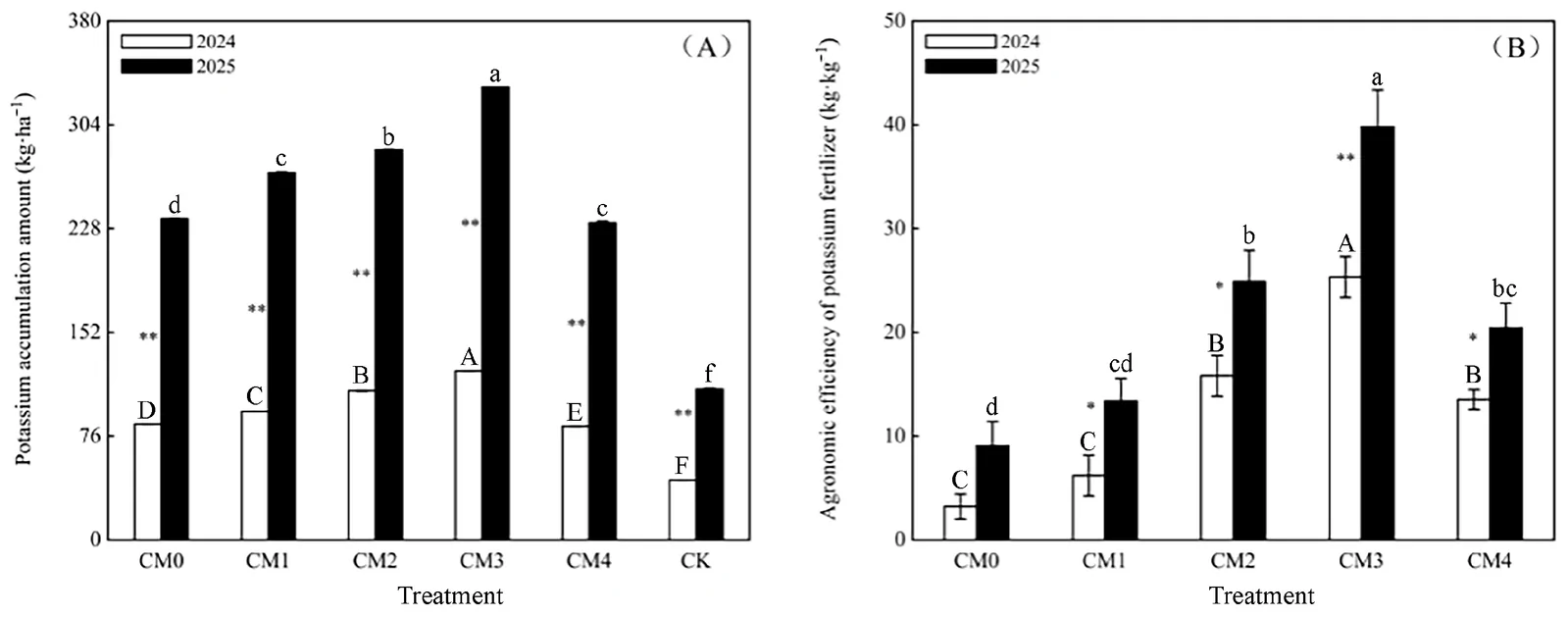
Plant potassium accumulation and agronomic potassium use efficiency of alfalfa under different fertilizer combinations. Figure (**A**) illustrates potassium accumulation, while Figure (**B**) presents the agronomic use efficiency of potassium fertilizer. Different uppercase letters indicate significant differences among different treatments in 2024 at the *p* < 0.05 level. Different lowercase letters indicate significant differences among different treatments in 2025 at the *p* < 0.05 level. * Significant difference for the same treatment between years at the *p* < 0.05 level; ** extremely significant difference for the same treatment between different years at the *p* < 0.01 level.

Varying ratios of organic to synthetic fertilizers differentially affected K accumulation and agronomic K use efficiency (AKUE) in alfalfa. As shown in Figure 3A, K accumulation was significantly higher under CM3 than under all other treatments in both years. Across all treatments, K accumulation increased highly significantly in 2025 relative to 2024 (*p* < 0.01). As shown in Figure 3B, AKUE was highest under CM3 in both years; no significant difference was detected between CM2 and CM4, CM0 exhibited the lowest value, although it did not differ significantly from CM1 in either year. Relative to 2024, AKUE in 2025 increased significantly under CM1, CM2, and CM4 (*p* < 0.05) and highly significantly under CM3 (*p* < 0.01).

In both 2024 and 2025, K accumulation and agronomic K use efficiency (AKUE) in alfalfa increased initially and then declined with increasing organic fertilizer substitution ratio (Table 3). In 2024, K accumulation in both cuttings was significantly higher under CM3 than under all other treatments, and all treatments differed significantly (*p* < 0.05). AKUE in both cuttings was also significantly higher under CM3 than under all other treatments, with no significant difference between CM0 and CM1 or between CM2 and CM4. In 2025, K accumulation across the four cuttings was significantly higher under CM3 than under all other treatments; only in the fourth cutting was no significant difference observed between CM0 and CM4, whereas all other treatments differed significantly (*p* < 0.05). AKUE across the four cuttings was highest under CM3. Specifically, under CM2 and CM3, AKUE generally declined with successive cuts, whereas the remaining treatments exhibited fluctuations.

#### 2.1.4. Response of Alfalfa Yield to Aboveground Nutrient Accumulation

**Figure 4:**
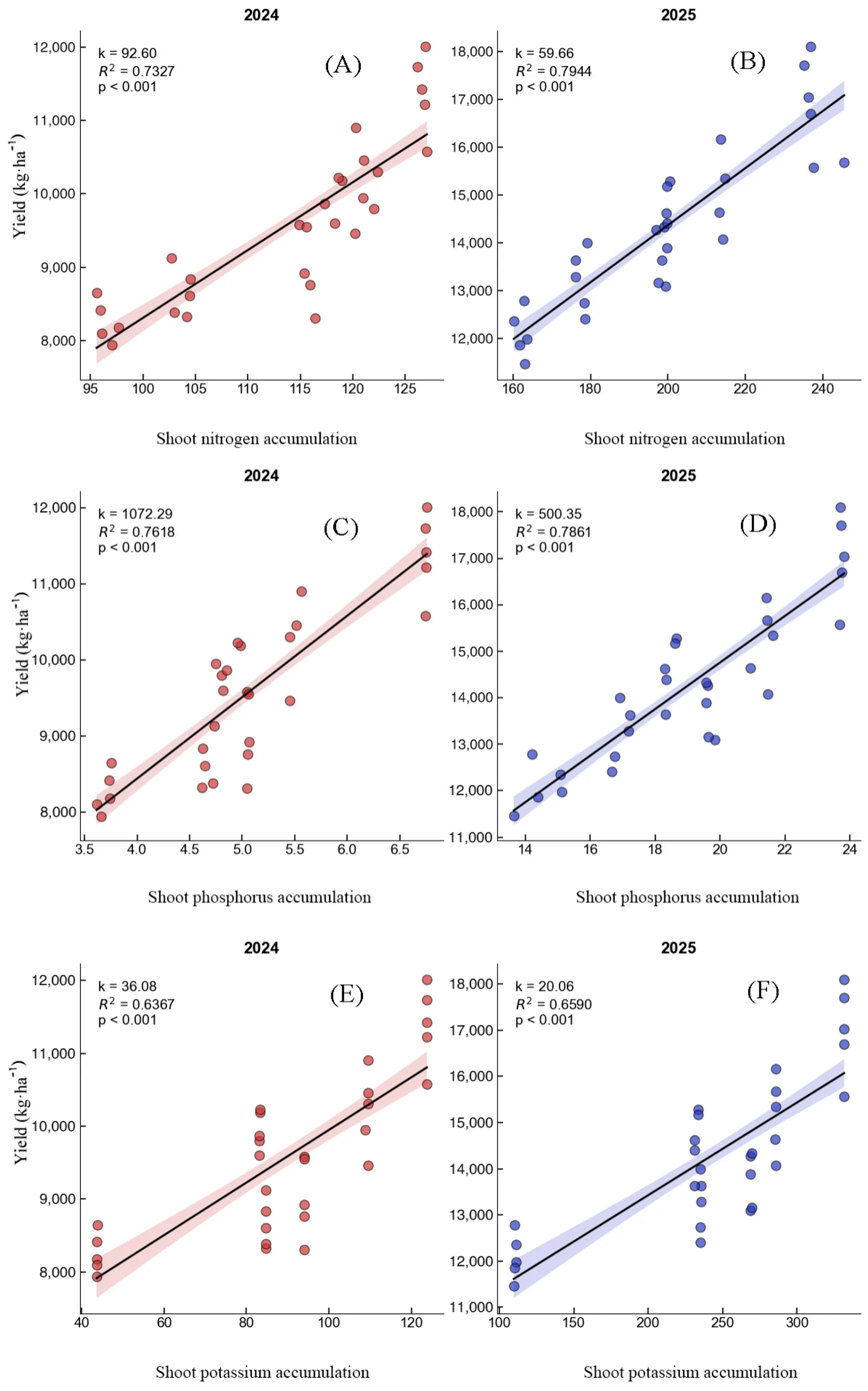
The relationship between alfalfa yield and plant nitrogen, phosphorus, and potassium accumulation. Figures (**A**,**B**) depict the relationships between crop yield and plant nitrogen accumulation in 2024 and 2025, while Figures (**C**,**D**) illustrate the correlations between crop yield and plant phosphorus accumulation across the two years. Similarly, Figures (**E**,**F**) present the associations between crop yield and plant potassium accumulation in 2024 and 2025.

Across experimental years, alfalfa yield exhibited a highly significant positive linear correlation (*p* < 0.001) with plant nitrogen, phosphorus, and potassium accumulation, demonstrating that nutrient accumulation is a critical determinant of alfalfa yield formation. Meanwhile, distinct differences existed in the explanatory capacity of individual nutrients for yield variation. As illustrated in Figure 4, nitrogen accumulation was strongly and positively correlated with alfalfa yield. In 2024, N accumulation explained a large proportion of yield variation (R^2^ = 0.7327, *p* < 0.001), and this correlation was further enhanced in 2025 (R^2^ = 0.7944, *p* < 0.001). Similarly, phosphorus accumulation showed a highly significant positive correlation with yield. The 2024 regression analysis yielded a coefficient of determination (R^2^) of 0.7618 for the P accumulation–yield relationship (*p* < 0.001), which was higher than that of potassium and slightly greater than that of nitrogen. This significant correlation persisted in 2025 (R^2^ = 0.7861, *p* < 0.001). Alfalfa yield increased significantly with elevated potassium accumulation in both experimental years. Potassium accumulation exhibited a significant positive linear relationship with yield in 2024 (R^2^ = 0.6367, *p* < 0.001), and this correlation was further strengthened in 2025 (R^2^ = 0.6590, *p* < 0.001). These findings indicated that although potassium accumulation substantially facilitated yield formation, its unit contribution to yield improvement declined relative to nitrogen and phosphorus. Comparing the regression relationships between yield and accumulation of N, P, and K revealed that N and P accumulation explained a greater proportion of yield variation than K accumulation, indicating that N and P play a more important role in alfalfa yield formation. In both years, yield increased in parallel with plant nutrient accumulation, suggesting that enhanced nutrient uptake and utilization contribute to alfalfa biomass accumulation and high yield. Notably, the strong fit between N and P accumulation and yield suggests that improving plant nutrient uptake efficiency may represent a viable pathway to high alfalfa yields, although the actual yield benefits require further validation. Future studies could employ multivariate approaches, such as principal component analysis (PCA) and structural equation modeling (SEM), to disentangle the relationships among yield, nutrient accumulation, and nutrient concentration.

## 3. Discussion

### 3.1. Effects of Combined Organic and Chemical Fertilizer Application on Nutrient Uptake Characteristics of Alfalfa

Hay yield is a key indicator of alfalfa forage productivity [32]. Fertilization strongly regulates alfalfa forage growth; rational fertilization supplies essential nutrients and facilitates robust plant development [33]. In this study, the optimized combined application of organic and chemical fertilizers markedly improved alfalfa hay yield, especially under the CM3 treatment (25% cow manure + 75% chemical fertilizer). Across 2024 and 2025, the CM3 treatment achieved alfalfa hay yields of 11,395.72 kg·hm^−2^ and 17,023.54 kg·hm^−2^, corresponding to yield increases of 38.03% and 40.85% relative to the control (CK), respectively. Rational organic–inorganic fertilizer blending effectively boosts alfalfa yield, consistent with findings from prior research. Variations in the optimal fertilizer ratio across studies are likely attributable to differences in soil properties and climatic conditions.

Efficient nutrient utilization is a key physiological basis for high alfalfa yields [34]. As a core constituent of proteins and enzymes, N, when efficiently assimilated, promotes root development and metabolic activity, thereby enhancing P and K uptake. P mediates energy metabolism via ATP synthesis and facilitates nutrient translocation, whereas K regulates stomatal function and enzyme activity to promote photosynthate accumulation and translocation. Collectively, these macronutrients regulate crop growth and yield formation [35,36,37].

At the soil scale, combined organic–inorganic fertilization has been shown to alter soil microbial community structure, enhance soil enzyme activity, accelerate nutrient mineralization and biogeochemical cycling, and ultimately improve crop N, P, and K use efficiency [38,39]. Multi-site field trials have further demonstrated that integrated organic–inorganic fertilization markedly increases hay yield and nutritional quality, reduces synthetic fertilizer input, and improves fertilizer use efficiency [40].

Regarding plant N dynamics, most studies have focused on annual grain crops such as winter wheat and have established that organic fertilizer substitution can enhance N accumulation, translocation, and use efficiency, although the optimal substitution rate and effect magnitude vary across studies. Long-term increased organic fertilization has been shown to enhance pre-anthesis N remobilization from vegetative organs by an average of 11.98% relative to inorganic fertilization alone [41]. A substitution rate of approximately 30% has been reported to optimize N translocation to reproductive organs [42], whereas substitution rates below 70% can mitigate N leaching and improve both N use efficiency and N uptake efficiency [43]. At a 20% substitution rate, winter wheat exhibited significant increases in aboveground N accumulation, pre-anthesis N translocation, and post-anthesis N accumulation; however, this rate did not markedly enhance the contributions of pre- and post-anthesis N translocation and accumulation to grain yield [44]. These findings indicate that although organic substitution generally improves plant N status, its effects on yield formation depend on crop type and the specific N translocation pathways involved.

The present results are generally consistent with these established patterns. The CM3 treatment (25% organic + 75% synthetic fertilizer) achieved the highest N, P, and K use efficiencies, with consistent trends across the three nutrients, suggesting strong synergistic regulation of crop nutrient uptake and utilization. Across all treatments, nutrient use efficiency exhibited a unimodal pattern—increasing then declining—with rising organic substitution rates, indicating that appropriate organic–inorganic fertilizer ratios optimize the synchronization between soil nutrient supply and crop nutrient demand, whereas excessive organic substitution may limit nutrient availability due to slower mineralization. Notably, the optimal substitution ratio in this study (25% organic + 75% synthetic) differs from that reported for winter wheat (20–30%), which may reflect differences between perennial forage legumes and annual grain crops in nutrient demand patterns, root traits, and symbiotic N fixation capacity. Additionally, nutrient accumulation and agronomic use efficiency were significantly higher in 2025 than in 2024 across all treatments, demonstrating that continuous organic fertilization gradually improves soil fertility and quality, enhances soil nutrient storage and microbially mediated nutrient cycling, and consequently increases crop nutrient utilization over time.

### 3.2. Variations in Yield Performance and Nutrient Utilization of Alfalfa

Forage yield is tightly coupled with aboveground nitrogen, phosphorus, and potassium accumulation [45]. Two consecutive years of field trials revealed that the CM3 treatment (25% cow manure + 75% chemical fertilizer) achieved the maximum hay yield and plant nutrient accumulation across all treatments. Plant nitrogen, phosphorus, and potassium accumulation exhibited a highly significant positive linear correlation with hay yield, indicating that nutrient accumulation plays an important role in biomass accumulation. Among these three macronutrients, nitrogen and phosphorus explained a greater proportion of yield variation than potassium. Nitrogen and phosphorus are indispensable for alfalfa protein synthesis, enzymatic reactions, and energy metabolism, directly regulating plant growth and photosynthate partitioning. Enhanced plant nitrogen accumulation facilitates protein synthesis, stimulates root development, and elevates enzyme activity, further promoting plant phosphorus and potassium uptake [34,46,47]. The core finding that greater nutrient accumulation drives higher forage yields aligns with prior research results. Wang Xiaoyu et al. [48] confirmed that integrated organic–inorganic fertilization markedly increases nitrogen accumulation and hay yield in alfalfa. Symanowicz and Skorupka also demonstrated that the combined application of nitrogen, phosphorus, and potassium fertilizers supplemented with the micronutrients iron and molybdenum (NPKFe_1_Mo_1_) significantly increased nitrogen accumulation and yield in alfalfa [30]. In wheat and maize cropping systems, partial organic fertilizer substitution also improves soil fertility and crop nutrient use efficiency [49]. Lu et al. [29] similarly reported that maintaining organic nitrogen substitution at 18–24% increases soil available nutrient contents and improves fertilizer use efficiency, sustaining stable and high crop yields. Integrated organic–inorganic fertilization synchronizes nutrient supply with crop nutrient demand dynamics, optimizes aboveground plant nutrient accumulation, and promotes yield improvement. Furthermore, two-year field trial results reveal the cumulative effects of organic fertilization: nutrient accumulation in alfalfa and fertilizer agronomic efficiency were both greater in 2025 than in 2024. As core indicators of soil quality and ecological function, soil microorganisms sustain soil ecosystem sustainability by mediating the synthesis, decomposition, and transformation of soil organic matter [50]. Long-term organic fertilization supplies soil microorganisms with abundant exogenous organic substrates and nutrients, stimulating microbial proliferation and shaping distinct rhizosphere microbial communities [51]. Combined organic and inorganic fertilization generally enhances the diversity and stability of soil microbial communities, enriches beneficial microbial taxa including Proteobacteria and Actinobacteria, and consequently elevates soil enzyme activity and nutrient availability [52]. Proteobacteria comprise rhizobia that establish symbiotic nitrogen-fixing relationships with alfalfa, facilitating plant nitrogen uptake; Bacteroidetes participate in soil organic carbon mineralization, driving terrestrial carbon and nitrogen cycling; and Actinobacteria decompose complex organic matter into low-molecular-weight nutrients, improve soil structure, and strengthen soil water and nutrient retention capacity [53,54]. The integrated application of organic and chemical fertilizers increases the relative abundance of these beneficial rhizosphere microbial communities, optimizes the rhizosphere microenvironment, modulates nutrient uptake by alfalfa, and improves fertilizer use efficiency, ultimately promoting alfalfa growth and forage quality [55]. These improvements can be attributed to progressive soil quality amelioration and enhanced microbial activity driven by continuous multi-year organic fertilization [56].

In summary, alfalfa hay yield exhibits a significant positive correlation with aboveground nutrient accumulation, underscoring the essential role of efficient nutrient uptake and utilization in high-yield production. An optimized fertilization regime, exemplified by the CM3 treatment, enhances alfalfa nitrogen, phosphorus, and potassium uptake, accelerates biomass accumulation, and sustains high forage yields. These findings demonstrate that scientifically balanced organic–inorganic fertilization improves alfalfa productivity while facilitating sustainable nutrient management in alfalfa cropping systems.

## 4. Materials and Methods

### 4.1. Study Area

The field experiment was carried out at the Sanping Farm Comprehensive Experimental Station of Xinjiang Agricultural University (87°21′13″ E, 43°56′58″ N). Situated on the southern edge of the Junggar Basin at an altitude of 580 m, the site features a temperate continental semi-arid climate with sufficient solar radiation. The regional mean annual temperature is 7.2 °C, with an annual sunshine duration of 2829.4 h, annual precipitation of 228.8 mm, and annual evaporation of 2647 mm. The frost-free period extends for 163 days, and the study area experiences a stable climate without extreme meteorological events. According to the World Reference Base for Soil Resources (IUSS Working Group WRB, 2014, 2022), the soil at the experimental site is classified as a Haplic Calcisol (Loamic). Prior to the experiment, the topsoil (0–20 cm) exhibited a silty loam texture, with sand, silt, and clay contents of 10.80%, 64.82%, and 24.39%, respectively. The basic physicochemical properties of the experimental soil and applied cow manure are presented in Table 4.

### 4.2. Experimental Design

This field experiment was arranged in a completely randomized block design with six fertilization treatments, including sole organic and synthetic fertilizer applications as well as their combined ratios: CM0 (100% cow manure), CM1 (75% cow manure + 25% synthetic fertilizer), CM2 (50% cow manure + 50% synthetic fertilizer), CM3 (25% cow manure + 75% synthetic fertilizer), CM4 (100% synthetic fertilizer), and CK (blank control). The fertilization ratio protocols adopted in this study were modified according to the method established by Xu Lulu et al. [57]. The total nitrogen application rate was maintained at a uniform level of 150 kg·ha^−1^ across all treatments [57]. The organic fertilizer utilized in this experiment was fully decomposed cow manure collected from the cattle ranch at Sanping Farm. All organic manure was applied as a one-time basal fertilizer before alfalfa sowing and regrowth during the 2024–2025 growing seasons. The synthetic fertilizer adopted for combined application was locally purchased urea, with a total nitrogen content of 46.67%, which was applied via a fertilizer tank. Differential topdressing schedules were implemented across the two experimental years. In 2024, a two-stage topdressing strategy was performed, with fertilizer supplied after each harvest at the late budding to early flowering stage of alfalfa. In 2025, the fertilization regime was adjusted to a three-stage topdressing pattern, with uniform topdressing application conducted following the harvest of the first three alfalfa cuttings.

The experimental material was ‘Xinmu No. 4’ alfalfa, provided by the College of Grassland Science, Xinjiang Agricultural University. The experiment included five replicates per treatment, resulting in a total of 30 plots (each 3 m × 4 m, with a 0.3 m row spacing); phosphorus and potassium were applied at 52.4 and 21.6 kg·ha^−1^, respectively, with application rates uniform across all plots [58]. The alfalfa seeding rate was 15 kg·hm^−2^, with manual row sowing at a depth of 1–2 cm. Soil nitrogen fertilization rates for each treatment were calculated based on the nitrogen content of synthetic chemical fertilizers and cow manure (Table 5).

Alfalfa was sown in April 2024. Two harvests were conducted in the first year: July 2024 (first harvest) and September 2024 (second harvest). Four harvests were performed in the second year: May 2025 (first harvest), July 2025 (second harvest), August 2025 (third harvest), and September 2025 (fourth harvest).

### 4.3. Measurement Parameters and Methods

#### Nutrient Accumulation and Utilization Efficiency in Plants

Alfalfa hay yield data utilized in the present study were retrieved from our previously published work entitled “The Combined Application of Organic Fertilizer and Chemical Fertilizer Increases Alfalfa Yield, Enhances Soil Nutrient Availability, and Improves Soil Biological Properties” in Agronomy [58], which was conducted at the identical experimental site (Table 6). Consistent data collection protocols and experimental backgrounds were adopted in the current study. On this basis, we further quantified and analyzed the accumulation and utilization efficiency of nitrogen, phosphorus, and potassium in alfalfa plants under diverse fertilization regimes.

Nutrient accumulation in alfalfa: The concentrations of N, P, and K in Medicago sativa samples were determined via the Kjeldahl method, vanadium-molybdenum yellow colorimetric method, and flame photometry, respectively. Plant nutrient accumulation (kg·ha^−1^) and fertilizer agronomic utilization efficiency (kg·kg^−1^) for N, P, and K were calculated based on the dry biomass of alfalfa plants under each fertilization regime.

The relevant calculation formulas are presented below:

N accumulation (kg·ha^−1^) = Treatment-specific dry biomass × Plant N concentration

N fertilizer agronomic efficiency (kg·kg^−1^) = (Yield of N fertilization treatment—Yield of blank control treatment)/N application rate

P accumulation (kg·ha^−1^) = Treatment-specific dry biomass × Plant P concentration

P fertilizer agronomic efficiency (kg·kg^−1^) = (Yield of P fertilization treatment—Yield of blank control treatment)/P application rate

K accumulation (kg·ha^−1^) = Treatment-specific dry biomass × Plant K concentration

K fertilizer agronomic efficiency (kg·kg^−1^) = (Yield of K fertilization treatment—Yield of blank control treatment)/K application rate

### 4.4. Statistical Analysis

Data processing was performed in Excel 2022, and statistical analyses were conducted in IBM SPSS 27.0. Prior to analysis, normality was assessed using the Shapiro–Wilk test and homogeneity of variances was verified using Levene’s test. One-way analysis of variance (ANOVA) was used to evaluate the effects of fertilization treatments within each year. When significant differences were detected, Tukey’s honestly significant difference (HSD) test was used for post hoc multiple comparisons (α = 0.05). Independent-samples *t*-tests were used to compare differences between years (2024 vs. 2025) within each fertilization treatment. Figures were generated using Origin 2024, and data are presented as means ± standard errors (*n* = 5).

## 5. Conclusions

The CM3 treatment (25% cow manure + 75% chemical fertilizer) significantly increased N, P, and K accumulation and nutrient use efficiency in alfalfa relative to the control (CK), 100% cow manure, and 100% chemical fertilizer treatments. Total N, P, and K accumulation exhibited marked interannual variation, with significantly higher values in 2025 than in 2024, reflecting a cumulative effect of repeated fertilization on soil nutrient supply and plant uptake over successive years. Combined organic–inorganic fertilization also enhanced the agronomic use efficiency of N, P, and K, with the strongest effect observed under CM3. Collectively, these findings indicate that an optimal organic-to-inorganic fertilizer ratio improves alfalfa yield and nutrient uptake and use efficiency under the present experimental conditions.

This study addressed only alfalfa yield and nutrient uptake and use efficiency; the mechanisms by which combined fertilization regulates nutrient utilization remain to be elucidated. Future research should investigate rhizosphere soil properties, microbial community dynamics, and symbiotic nitrogen fixation in root nodules across fertilization regimes. Furthermore, long-term field experiments are needed to assess the persistence and stability of combined organic–inorganic fertilization effects, providing a more robust theoretical basis for rational alfalfa fertilization management in arid regions.

## Figures and Tables

**Table 1:** Nitrogen utilization efficiency of alfalfa plants under the combination of organic fertilizer and chemical fertilizer.

Item	Treatment	2024		2025			
		First Crop	Second Crop	First Crop	Second Crop	Third Crop	Fourth Crop
Nitrogen accumulation/(kg·ha^−1^)	CM0	55.25 ± 0.57D	48.50 ± 0.34E	75.25 ± 0.57e	51.50 ± 0.34d	28.25 ± 0.57bc	22.65 ± 0.13e
	CM1	60.20 ± 0.49C	55.41 ± 0.36D	80.00 ± 0.58d	56.54 ± 0.18c	35.40 ± 0.51ab	26.57 ± 0.18c
	CM2	64.40 ± 0.94B	56.57 ± 0.39B	86.40 ± 0.38b	62.45 ± 0.32b	42.88 ± 6.24a	28.51 ± 0.21b
	CM3	68.36 ± 0.26A	58.33 ± 0.16A	90.36 ± 0.26a	65.44 ± 0.32a	40.36 ± 0.26a	40.33 ± 0.26a
	CM4	63.94 ± 0.33C	55.08 ± 0.65C	82.94 ± 0.53c	56.35 ± 0.19c	35.10 ± 0.09ab	25.20 ± 0.13d
	CK	50.93 ± 0.58E	45.52 ± 0.43F	70.69 ± 0.63f	45.89 ± 0.54e	25.04 ± 0.57c	20.62 ± 0.29e
Agronomic nitrogen fertilizer use efficiency/(kg·kg^−1^)	CM0	2.07 ± 0.52C	0.58 ± 0.52C	2.07 ± 0.52c	4.40 ± 1.40b	0.58 ± 0.52c	0.44 ± 0.17c
	CM1	3.49 ± 0.72C	1.63 ± 0.90C	3.49 ± 0.72c	5.45 ± 1.00ab	1.63 ± 0.90c	0.51 ± 0.28c
	CM2	7.78 ± 0.85B	5.28 ± 0.83B	7.79 ± 0.85b	5.58 ± 0.62ab	5.28 ± 0.83b	1.96 ± 0.26b
	CM3	12.07 ± 0.65A	8.87 ± 0.98A	12.07 ± 0.66a	8.87 ± 0.98a	8.87 ± 0.98a	3.12 ± 0.31a
	CM4	6.07 ± 0.42B	5.13 ± 0.44B	6.07 ± 0.42b	4.09 ± 0.58b	5.13 ± 0.44b	1.60 ± 0.13b
	CK						

Note: Different uppercase letters indicate significant differences among different treatments for the same cutting in alfalfa 2024 at the *p* < 0.05 level; different lowercase letters indicate significant differences among different treatments for the same cutting in alfalfa 2025 at the *p* < 0.05 level.

**Table 2:** Phosphorus utilization efficiency of alfalfa plants under the combination application of organic fertilizer and chemical fertilizer.

Item	Treatment	2024		2025			
		FIRST CROP	Second Crop	First Crop	Second Crop	Third Crop	Fourth Crop
Phosphorus accumulation/(kg·ha^−1^)	CM0	2.65 ± 0.01D	2.02 ± 0.01D	6.29 ± 0.03d	4.46 ± 0.01e	3.60 ± 0.04e	2.59 ± 0.01c
	CM1	2.84 ± 0.01C	2.14 ± 0.01C	7.46 ± 0.01b	4.96 ± 0.05c	4.45 ± 0.01c	2.79 ± 0.01bc
	CM2	2.98 ± 0.07B	2.36 ± 0.07B	7.55 ± 0.02b	6.11 ± 0.02b	4.78 ± 0.07b	2.93 ± 0.07b
	CM3	3.68 ± 0.01A	3.06 ± 0.04A	8.43 ± 0.01a	6.60 ± 0.04a	5.07 ± 0.03a	3.63 ± 0.01a
	CM4	2.75 ± 0.01C	2.13 ± 0.01C	7.08 ± 0.05c	4.62 ± 0.02d	4.04 ± 0.01d	2.70 ± 0.01bc
	CK	2.16 ± 0.01E	1.53 ± 0.01E	5.86 ± 0.03e	4.04 ± 0.05f	3.02 ± 0.05f	1.56 ± 0.24d
Agronomic phosphorus fertilizer use efficiency/(kg·kg^−1^)	CM0	3.46 ± 0.87C	0.97 ± 0.88C	3.46 ± 0.87c	7.33 ± 2.34b	0.97 ± 0.88c	0.74 ± 0.28c
	CM1	5.82 ± 1.20C	2.72 ± 1.50C	5.82 ± 1.20c	9.07 ± 1.66ab	2.72 ± 1.50c	0.85 ± 0.47c
	CM2	12.98 ± 1.42B	8.80 ± 1.38B	12.98 ± 1.42b	9.29 ± 1.04ab	8.80 ± 1.39b	3.27 ± 0.44b
	CM3	20.11 ± 0.10A	14.78 ± 1.63A	20.11 ± 1.10a	14.78 ± 1.63a	14.78 ± 1.63a	5.19 ± 0.52a
	CM4	10.12 ± 0.70B	8.54 ± 0.73B	10.12 ± 0.70b	6.81 ± 2.52b	8.54 ± 0.73b	2.67 ± 0.22b
	CK						

Note: Different uppercase letters indicate significant differences among different treatments for the same cutting in alfalfa 2024 at the *p* < 0.05 level; different lowercase letters indicate significant differences among different treatments for the same cutting in alfalfa 2025 at the *p* < 0.05 level.

**Table 3:** Potassium utilization efficiency of alfalfa plants under the combination application of organic fertilizer and chemical fertilizer.

Item	Treatment	2024		2025			
		First Crop	Second Crop	First Crop	Second Crop	Third Crop	Fourth Crop
Potassium accumulation/(kg·ha^−1^)	CM0	51.65 ± 0.02D	33.02 ± 0.02E	82.29 ± 0.08e	61.66 ± 0.10d	53.60 ± 0.10d	37.60 ± 0.02d
	CM1	55.84 ± 0.01C	38.21 ± 0.01C	87.46 ± 0.01c	79.96 ± 0.12c	58.47 ± 0.54c	43.12 ± 0.01c
	CM2	62.98 ± 0.16B	46.36 ± 0.16B	92.55 ± 0.05b	83.14 ± 0.06b	65.13 ± 0.17b	44.96 ± 0.16b
	CM3	69.69 ± 0.01A	54.06 ± 0.01A	108.44 ± 0.01a	91.60 ± 0.04a	73.07 ± 0.03a	58.63 ± 0.01a
	CM4	50.05 ± 0.04E	33.13 ± 0.04D	83.08 ± 0.12d	60.62 ± 0.05e	50.90 ± 1.07e	37.70 ± 0.04d
	CK	25.16 ± 0.03F	18.54 ± 0.03F	35.86 ± 0.08f	31.04 ± 0.11f	26.02 ± 0.11f	17.56 ± 0.54e
Agronomic potassium fertilizer use efficiency/(kg·kg^−1^)	CM0	2.51 ± 0.63C	0.71 ± 0.64C	2.51 ± 0.63c	5.32 ± 1.70b	0.71 ± 0.63c	0.54 ± 0.20c
	CM1	4.23 ± 0.87C	1.97 ± 1.09C	4.23 ± 0.87c	6.59 ± 1.21ab	1.97 ± 1.09c	0.62 ± 0.34c
	CM2	9.42 ± 1.03B	6.39 ± 1.00B	9.42 ± 1.03b	6.74 ± 0.75ab	6.39 ± 1.00b	2.37 ± 0.32b
	CM3	14.59 ± 0.79A	10.73 ± 1.87A	14.60 ± 0.79a	10.73 ± 1.18a	10.73 ± 1.18a	3.77 ± 0.38a
	CM4	7.35 ± 0.51B	6.02 ± 0.53B	7.35 ± 0.51b	4.94 ± 1.83b	6.20 ± 0.53b	1.94 ± 0.16b
	CK						

Note: Different uppercase letters indicate significant differences among different treatments for the same cutting in alfalfa 2024 at the *p* < 0.05 level; different lowercase letters indicate significant differences among different treatments for the same cutting in alfalfa 2025 at the *p* < 0.05 level.

**Table 4:** Physical and chemical properties of organic fertilizer and cultivated soil.

Type	pH	TN/(g/kg)	TP/(g/kg)	TK/(g/kg)	AN/(mg/kg)	AP/(mg/kg)	AK/(mg/kg)	SOM/(g/kg)
Soil	7.91	0.71	0.42	13.22	36.50	10.25	123.15	13.28
Cow dung	8.12	0.46	0.89	1.24	65.23	26.71	132.00	42.20

Note: TN: total nitrogen; TP: total phosphorus; TK: total potassium; AN: alkaline nitrogen; AP: available phosphorus; AK: available potassium; SOM: organic matter.

**Table 5:** The soil fertilization rates for each treatment.

Fertilizer Application Amount	Cow Dung/t·ha^−1^	Mineral Fertilizer (Urea), kg N ha^−1^
CM0	32.45	0.00
CM1	24.33	36.80
CM2	16.22	73.60
CM3	8.11	110.40
CM4	0.00	147.20
CK	0.00	0.00

**Table 6:** Hay yield and underground biomass of alfalfa under different fertilizer application treatments.

Soil Layer	Treatments	Years	
		2024	2025
Hay Yield/t·ha^−1^	CM0	8.65 ± 0.15CD **	13.21 ± 0.29d
	CM1	9.02 ± 0.24CD **	13.75 ± 0.27cd
	CM2	10.22 ± 0.24B **	15.18 ± 0.37b
	CM3	11.40 ± 0.24A **	17.02 ± 0.44a
	CM4	9.94 ± 0.12B **	14.98 ± 0.33bc
	CK	8.26 ± 0.12D **	12.09 ± 0.23e

Note: Different uppercase letters indicate significant differences among different treatments in 2024 at the *p* < 0.05 level. Different lowercase letters indicate significant differences among different treatments in 2025 at the *p* < 0.05 level. ** extremely significant difference for the same treatment between different years at the *p* < 0.01 level.

## Data Availability

The data that support the findings of this study are available from the corresponding author, L.W., upon reasonable request.
